# Structural analysis of bacteriophage T4 DNA replication: a review in the Virology Journal series on *bacteriophage T4 and its relatives*

**DOI:** 10.1186/1743-422X-7-359

**Published:** 2010-12-03

**Authors:** Timothy C Mueser, Jennifer M Hinerman, Juliette M Devos, Ryan A Boyer, Kandace J Williams

**Affiliations:** 1 Department of Chemistry, University of Toledo, Toledo OH, USA; 2 Department of Molecular Genetics, Biochemistry & Microbiology, University of Cincinnati College of Medicine, Cincinnati, OH, USA; 3 European Molecular Biology Laboratory, Grenoble Outstation, Grenoble, France; 4 Texas State University, San Marcos, TX, USA; 5 Department of Biochemistry and Cancer Biology, University of Toledo College of Medicine, Toledo OH, USA

## Abstract

The bacteriophage T4 encodes 10 proteins, known collectively as the replisome, that are responsible for the replication of the phage genome. The replisomal proteins can be subdivided into three activities; the replicase, responsible for duplicating DNA, the primosomal proteins, responsible for unwinding and Okazaki fragment initiation, and the Okazaki repair proteins. The replicase includes the gp43 DNA polymerase, the gp45 processivity clamp, the gp44/62 clamp loader complex, and the gp32 single-stranded DNA binding protein. The primosomal proteins include the gp41 hexameric helicase, the gp61 primase, and the gp59 helicase loading protein. The RNaseH, a 5' to 3' exonuclease and T4 DNA ligase comprise the activities necessary for Okazaki repair. The T4 provides a model system for DNA replication. As a consequence, significant effort has been put forth to solve the crystallographic structures of these replisomal proteins. In this review, we discuss the structures that are available and provide comparison to related proteins when the T4 structures are unavailable. Three of the ten full-length T4 replisomal proteins have been determined; the gp59 helicase loading protein, the RNase H, and the gp45 processivity clamp. The core of T4 gp32 and two proteins from the T4 related phage RB69, the gp43 polymerase and the gp45 clamp are also solved. The T4 gp44/62 clamp loader has not been crystallized but a comparison to the *E. coli *gamma complex is provided. The structures of T4 gp41 helicase, gp61 primase, and T4 DNA ligase are unknown, structures from bacteriophage T7 proteins are discussed instead. To better understand the functionality of T4 DNA replication, in depth structural analysis will require complexes between proteins and DNA substrates. A DNA primer template bound by gp43 polymerase, a fork DNA substrate bound by RNase H, gp43 polymerase bound to gp32 protein, and RNase H bound to gp32 have been crystallographically determined. The preparation and crystallization of complexes is a significant challenge. We discuss alternate approaches, such as small angle X-ray and neutron scattering to generate molecular envelopes for modeling macromolecular assemblies.

## Bacteriophage T4 DNA Replication

The semi-conservative, semi-discontinuous process of DNA replication is conserved in all life forms. The parental anti-parallel DNA strands are separated and copied following hydrogen bonding rules for the keto form of each base as proposed by Watson and Crick [1]. Progeny cells therefore inherit one parental strand and one newly synthesized strand comprising a new duplex DNA genome. Protection of the integrity of genomic DNA is vital to the survival of all organisms. In a masterful dichotomy, the genome encodes proteins that are also the caretakers of the genome. RNA can be viewed as the evolutionary center of this juxtaposition of DNA and protein. Viruses have also played an intriguing role in the evolutionary process, perhaps from the inception of DNA in primordial times to modern day lateral gene transfer. Simply defined, viruses are encapsulated genomic information. Possibly an ancient encapsulated virus became the nucleus of an ancient prokaryote, a symbiotic relationship comparable to mitochondria, as some have recently proposed [2-4]. This early relationship has evolved into highly complex eukaryotic cellular processes of replication, recombination and repair requiring multiple signaling pathways to coordinate activities required for the processing of complex genomes. Throughout evolution, these processes have become increasing complicated with protein architecture becoming larger and more complex. Our interest, as structural biologists, is to visualize these proteins as they orchestrate their functions, posing them in sequential steps to examine functional mechanisms. Efforts to crystallize proteins and protein:DNA complexes are hampered for multiple reasons, from limited solubility and sample heterogeneity to the fundamental lack of crystallizability due to the absence of complimentary surface contacts required to form an ordered lattice. For crystallographers, the simpler organisms provide smaller proteins with greater order which have a greater propensity to crystallize. Since the early days of structural biology, viral and prokaryotic proteins were successfully utilized as model systems for visualizing biological processes. In this review, we discuss our current progress to complete a structural view of DNA replication using the viral proteins encoded by bacteriophage T4 or its relatives.

DNA replication initiation is best exemplified by interaction of the *E. coli *DnaA protein with the *OriC *sequence which promotes DNA unwinding and the subsequent bi-directional loading of DnaB, the replicative helicase [5]. Assembly of the replication complex and synthesis of an RNA primer by DnaG initiates the synthesis of complimentary DNA polymers, comprising the elongation phase. The bacteriophage T4 encodes all of the proteins essential for its DNA replication. Table 1 lists these proteins, their functions and corresponding T4 genes. Through the pioneering work of Nossal, Alberts, Konigsberg, and others, the T4 DNA replication proteins have all been isolated, analyzed, cloned, expressed, and purified to homogeneity. The replication process has been reconstituted, using purified recombinant proteins, with velocity and accuracy comparable to *in vivo *reactions [6]. Initiation of phage DNA replication within the T4-infected cell is more complicated than for the *E. coli *chromosome, as the multiple circularly permuted linear copies of the phage genome appear as concatemers with homologous recombination events initiating strand synthesis during middle and late stages of infection ([7], see Kreuzer and Brister this series).

**Table 1:** DNA Replication Proteins Encoded by Bacteriophage T4

Protein	Function
*Replicase*	
gp43 DNA polymerase	DNA directed 5' to 3' DNA polymerase
gp45 protein	Polymerase clamp enhances processivity of gp43 polymerase and RNase H
gp44/62 protein	clamp loader utilizes ATP to open and load the gp45 clamp
gp32 protein	cooperative single stranded DNA binding protein assists in unwinding duplex
*Primosome*	
gp41 helicase	processive 5' to 3' replicative helicase
gp61 primase	DNA dependent RNA polymerase generates lagging strand RNA pentamer primers in concert with gp41 helicase
gp59 protein	helicase assembly protein required for loading the gp41 helicase in the presence of gp32 protein
*Lagging strand repair*	
RNase H	5' to 3' exonuclease cleaves Okazaki RNA primers
gp30 DNA ligase	ATP-dependent ligation of nicks after lagging strand gap repair

The bacteriophage T4 replisome can be subdivided into two components, the DNA replicase and the primosome. The DNA replicase is composed of the gene 43-encoded DNA polymerase (gp43), the gene 45 sliding clamp (gp45), the gene 44 and 62 encoded ATP-dependent clamp loader complex (gp44/62), and the gene 32 encoded single-stranded DNA binding protein (gp32) [6]. The gp45 protein is a trimeric, circular molecular clamp that is equivalent to the eukaryotic processivity factor, proliferating cell nuclear antigen (PCNA) [8]. The gp44/62 protein is an accessory protein required for gp45 loading onto DNA [9]. The gp32 protein assists in the unwinding of DNA and the gp43 DNA polymerase extends the invading strand primer into the next genome, likely co-opting the *E. coli *gyrase (topo II) to reduced positive supercoiling ahead of the polymerase [10]. The early stages of elongation involves replication of the leading strand template in which gp43 DNA polymerase can continuously synthesize a daughter strand in a 5' to 3' direction. The lagging strand requires segmental synthesis of Okazaki fragments which are initiated by the second component of the replication complex, the primosome. This T4 replicative complex is composed of the gp41 helicase and the gp61 primase, a DNA directed RNA polymerase [11]. The gp41 helicase is a homohexameric protein that encompasses the lagging strand and traverses in the 5' to 3' direction, hydrolyzing ATP as it unwinds the duplex in front of the replisome [12]. Yonesaki and Alberts demonstrated that gp41 helicase cannot load onto replication forks protected by the gp32 protein single-stranded DNA binding protein [13,14]. The T4 gp59 protein is a helicase loading protein comparable to *E. coli *DnaC and is required for the loading of gp41 helicase if DNA is preincubated with the gp32 single-stranded DNA binding protein [15]. We have shown that the gp59 protein preferentially recognizes branched DNA and Holliday junction architectures and can recruit gp32 single-strand DNA binding protein to the 5' arm of a short fork of DNA [16,17]. The gp59 helicase loading protein also delays progression of the leading strand polymerase, allowing for the assembly and coordination of lagging strand synthesis. Once gp41 helicase is assembled onto the replication fork by gp59 protein, the gp61 primase synthesizes an RNA pentaprimer to initiate lagging strand Okazaki fragment synthesis. It is unlikely that the short RNA primer, in an A-form hybrid duplex with template DNA, would remain annealed in the absence of protein, so a hand-off from primase to either gp32 protein or gp43 polymerase is probably necessary [18].

**Figure 1:**
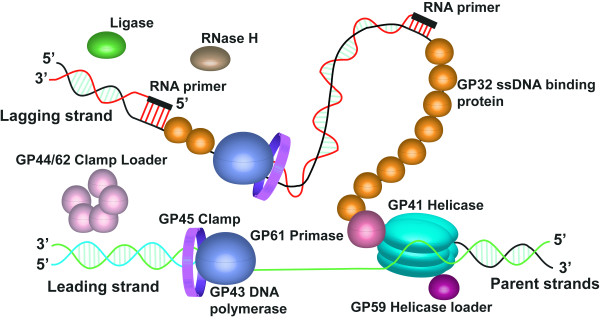
**A cartoon model of leading and lagging strand DNA synthesis by the Bacteriophage T4 Replisome**. The replicase proteins include the gp43 DNA polymerase, responsible for leading and lagging strand synthesis, the gp45 clamp, the ring shaped processivity factor involved in polymerase fidelity, and gp44/62 clamp loader, an AAA + ATPase responsible for opening gp45 for placement and removal on duplex DNA. The primosomal proteins include the gp41 helicase, a hexameric 5' to 3' ATP dependent DNA helicase, the gp61 primase, a DNA dependent RNA polymerase responsible for synthesis of primers for lagging strand synthesis, the gp32 single stranded DNA binding protein, responsible for protection of single stranded DNA created by gp41 helicase activity, and the gp59 helicase loading protein, responsible for the loading of gp41 helicase onto gp32 protected ssDNA. Repair of Okazaki fragments is accomplished by the RNase H, a 5' to 3' exonuclease, and gp30 ligase, the ATP dependent DNA ligase. Leading and lagging strand synthesis is coordinated by the replisome. Lagging strand primer extension and helicase progression lead to the formation of a loop of DNA extending from the replisome as proposed in the "trombone" model [21].

Both the leading and lagging strands of DNA are synthesized by the gp43 DNA polymerase simultaneously, similar to most prokaryotes. Okazaki fragments are initiated stochastically every few thousand bases in prokaryotes (eukaryotes have slower pace polymerases with primase activity every few hundred bases) [19]. The lagging strand gp43 DNA polymerase is physically coupled to the leading strand gp43 DNA polymerase. This juxtaposition coordinates synthesis while limiting the generation of single-stranded DNA[20]. As synthesis progresses, the lagging strand duplex extrude from the complex creating a loop, or as Alberts proposed, a trombone shape (Figure 1) [21]. Upon arrival at the previous Okazaki primer, the lagging strand gp43 DNA polymerase halts, releases the newly synthesized duplex, and rebinds to a new gp61 generated primer. The RNA primers are removed from the lagging strands by the T4 *rnh *gene encoded RNase H, assisted by gp32 single-strand binding protein if the polymerase has yet to arrive or by gp45 clamp protein if gp43 DNA polymerase has reached the primer prior to processing [22-24]. For this latter circumstance, the gap created by RNase H can be filled either by reloading of gp43 DNA polymerase or by *E. coli *Pol I [25]. The *rnh*^- ^phage are viable indicating that *E. coli *Pol I 5' to 3' exonuclease activity can substitute for RNase H [25]. Repair of the gap leaves a single-strand nick with a 3' OH and a 5' monophosphate, repaired by the gp30 ATP-dependent DNA ligase; better known as T4 ligase [26]. Coordination of each step involves molecular interactions between both DNA and the proteins discussed above. Elucidation of the structures of DNA replication proteins reveals the protein folds and active sites as well as insight into molecular recognition between the various proteins as they mediate transient interactions.

## Crystal Structures of the T4 DNA Replication Proteins

**Figure 2:**
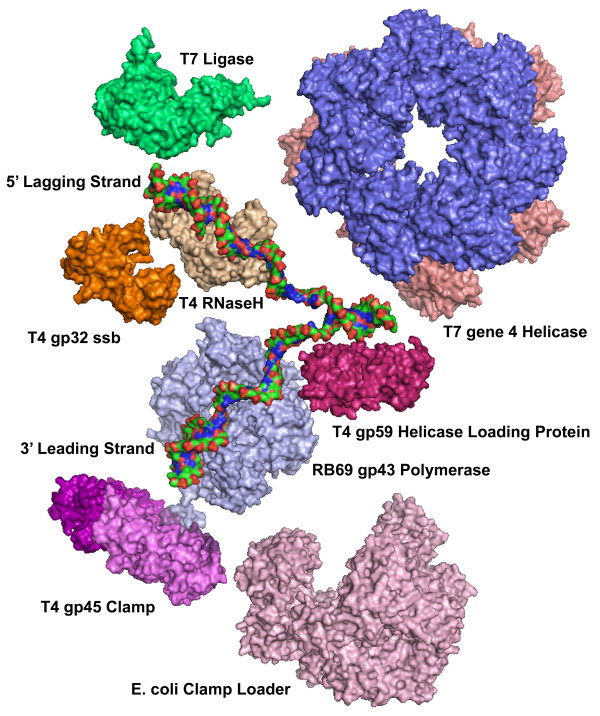
**The molecular models, rendered to scale, of a DNA replication fork**. Structures of four of ten T4 proteins are known; the RNase H (tan), the gp59 helicase loading protein (rose), the gp45 clamp (magenta), and the gp32 ssb (orange). Two additional structures from RB69, a T4 related phage, have also been completed; the RB69 gp43 polymerase (light blue) and the gp45 clamp (not shown). The E. coli clamp loader (γ complex) (pink) is used here in place of the T4 gp44/62 clamp loader, and two proteins from bacteriophage T7, T7 ligase (green) and T7 gene 4 helicase-primase (blue/salmon) are used instead of T4 ligase, and gp41/gp61, respectively.

In the field of protein crystallography, approximately one protein in six will form useful crystals. However, the odds frequently appear to be inversely proportional to overall interest in obtaining the structure. Our first encounter with T4 DNA replication proteins was a draft of Nancy Nossal's review "The Bacteriophage T4 DNA Replication Fork" subsequently published as Chapter 5 in the 1994 edition of "Molecular Biology of Bacteriophage T4" [6]. At the beginning of our collaboration (NN with TCM), the recombinant T4 replication system had been reconstituted and all 10 proteins listed in Table 1 were available [27]. Realizing the low odds for successful crystallization, all 10 proteins were purified and screened. Crystals were observed for 4 of the 10 proteins; gp43 DNA polymerase, gp45 clamp, RNase H, and gp59 helicase loading protein. We initially focused our efforts on solving the RNase H crystal structure, a protein first described by Hollingsworth and Nossal [24] and subsequently determined to be more structurally similar to the FEN-1 5' to 3' exonuclease family, rather than RNase H proteins [28]. The second crystal we observed was of the gp59 helicase loading protein first described by Yonesaki and Alberts [13,14]. To date, T4 RNase H, gp59 helicase loading protein, and gp45 clamp are the only full length T4 DNA replication proteins for which structures are available [17,28,29]. When proteins do not crystallize, there are several approaches to take. One avenue is to search for homologous organisms, such as the T4 related genome sequences ([30];Petrov et al. this series) in which the protein function is the same but the surface residues may have diverged sufficiently to provide compatible lattice interactions in crystals. For example, the Steitz group has solved two structures from a related bacteriophage, the RB69 gp43 DNA polymerase and gp45 sliding clamp [31,32]. Our efforts with a more distant relative, the vibriophage KVP40, unfortunately yielded insoluble proteins. Another approach is to cleave flexible regions of proteins using either limited proteolysis or mass spectrometry fragmentation. The stable fragments are sequenced using mass spectrometry and molecular cloning is used to prepare core proteins for crystal trials. Again, the Steitz group successfully used proteolysis to solve the crystal structure of the core fragment of T4 gp32 single-stranded DNA binding protein (ssb) [33]. This accomplishment has brought the total to five complete or partial structures of the ten DNA replication proteins from T4 or related bacteriophage. To complete the picture, we must rely on other model systems, the bacteriophage T7 and *E. coli *(Figure 2). We provide here a summary of our collaborative efforts with the late Dr. Nossal, and also the work of many others, that, in total, has created a pictorial view of prokaryotic DNA replication. A list of proteins of the DNA replication fork along with the relevant protein data bank (PDB) numbers is provided in Table 2.

**Table 2:** Proteins of the DNA Replication Fork and Protein Database (pdb) reference numbers.

	T4 and Related Phage	T7 Phage	*E. coli*	Eukaryotes
Replicative DNA polymerase	gp43 polymerase *(pdb *1ig9, 1clq, 1noy, 1ih7)	Gene 5 Polymerase *(pdb *1t7p, 1skr, 1t8e, 1x9m)	Pol III (αεθ) *(pdb *2hnh, 1ido)	Pol δ (subunits p125, p50, p66, p12) *(pdb *3e0j)
Sliding clamp	gp45 protein *(pdb *1czd, 1b8h)	E. coli Thioredoxin *(pdb *1t7p, 1skr)	β *(pdb *2pol)	PCNA *(pdb *1plr, 1axc, 1ul1)
Clamp loader	gp44/62 protein		γ complex (γδδ'ψχ) *(pdb *1jr3, 1jqj, 3glf)	RF-C (subunits p140, p40, p38, p37, p36) *(pdb *1sxj)
ssDNA binding protein	gp32 protein *(pdb *1gpc)	Gene 2.5 protein *(pdb *1je5)	SSB *(pdb *1qvc)	RP-A (subunits p14, p32, p70) *(pdb *1fgu, 2b29, 1jmc, 1l1o, 2pi2)
Replicative helicase	gp41 helicase	Gene 4 helicase *(pdb *1e0j, 1e0k, 1q57)	DnaB *(pdb *1b79, 2r6a)	MCM *(pdb *3f9v, 1ltl)
helicase assembly protein	gp59 protein *(pdb *1c1k)		DnaC, PriA *(pdb *3ec2)	
primase	gp61 primase	Gene 4 primase *(pdb *1nui, 1q57)	DnaG *(pdb *1dd9, 3b39, 2r6c)	Pol α/primase *(pdb *3flo)
5' to 3' Exonuclease	RNase H *(pdb *1tfr, 2ihn)		Pol I N-domain	FEN-1, RNase H1 *(pdb *1ul1, 2qk9)
DNA ligase 1	T4 ligase (gp30)	T7 ligase *(pdb *1a0i)	DNA ligase (pdb 2owo)	DNA ligase I

### Replicase Proteins

#### Gene 43 DNA Polymerase

**Figure 3:**
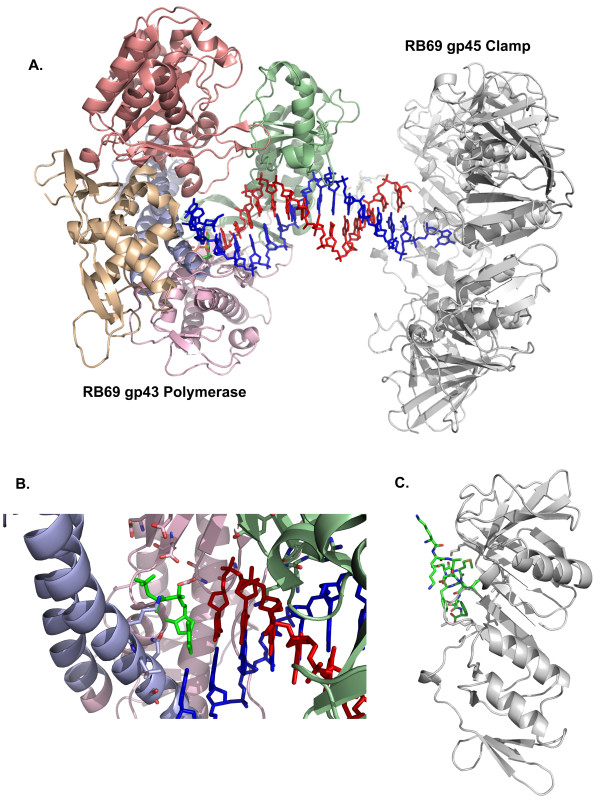
**The gp43 DNA polymerase from bacteriophage RB69 has been solved in complex with a DNA primer/template.** The gp45 clamp from RB69 has been solved in complex with a synthetic peptide containing the PIP box motif. A.) The RB69 gp43 polymerase in complex with DNA is docked to the RB69 gp45 clamp with the duplex DNA aligned with the central opening of gp45 (gray). The N-terminal domain (tan), the 3' - 5' editing exonuclease (salmon), the palm domain (pink), the fingers domain (light blue), and thumb domain (green comprise the DNA polymerase. The C-terminal residues extending from the thumb domain contain the PCNA interacting protein box motif (PIP box) shown docked to the 45 clamp. B.) The active site of the gp43 polymerase displays the template base to the active site with the incoming dNTP base paired and aligned for polymerization. C.) The C-terminal PIP box peptide (green) is bound to a subunit of the RB69 gp45 clamp (gray).

The T4 gp43 DNA polymerase (gi:118854, NP_049662), an 898 amino acid residue protein related to the Pol B family, is used in both leading and lagging strand DNA synthesis. The Pol B family includes eukaryotic pol α, δ, and ε. The full length T4 enzyme and the exo^- ^mutant (D219A) have been cloned, expressed and purified [34,35]. While the structure of the T4 gp43 DNA polymerase has yet to be solved, the enzyme from the RB69 bacteriophage has been solved individually (PDB 1waj) and in complex with a primer template DNA duplex (PDB 1ig9, Figure 3A) [32,36]. The primary sequence alignment reveals that the T4 gp43 DNA polymerase is 62% identical and 74% similar to RB69 gp43 DNA polymerase, a 903 residue protein [37,38].

*E. coli *Pol I, the first DNA polymerase discovered by Kornberg, has three domains, an N-terminal 5' to 3' exonuclease (cleaved to create the Klenow fragment), a 3' to 5' editing exonuclease domain, and a C-terminal polymerase domain [5]. The structure of the *E. coli *Pol I Klenow fragment was described through anthropomorphic terminology of fingers, palm, and thumb domains [39,40]. The RB69 gp43 DNA polymerase has two active sites, the 3' to 5' exonuclease (residues 103 - 339) and the polymerase domain (residues 381 - 903), comparable to Klenow fragment domains [41]. The gp43 DNA polymerase also has an N-terminal domain (residues 1 - 102 and 340 - 380) and a C-terminal tail containing a PCNA interacting peptide (PIP box) motif (residues 883 - 903) that interacts with the 45 sliding clamp protein. The polymerase domain contains a fingers subunit (residues 472 - 571) involved in template display (Ser 565, Lys 560, amd Leu 561) and NTP binding (Asn 564) and a palm domain (residues 381 - 471 and 572 - 699) which contains the active site, a cluster of aspartate residues (Asp 411, 621, 622, 684, and 686) that coordinates the two divalent active site metals (Figure 3B). The T4 gp43 DNA polymerase appears to be active in a monomeric form, however it has been suggested that polymerase dimerization is necessary to coordinate leading and lagging strand synthesis [6,20].

#### Gene 45 Clamp

**Figure 4:**
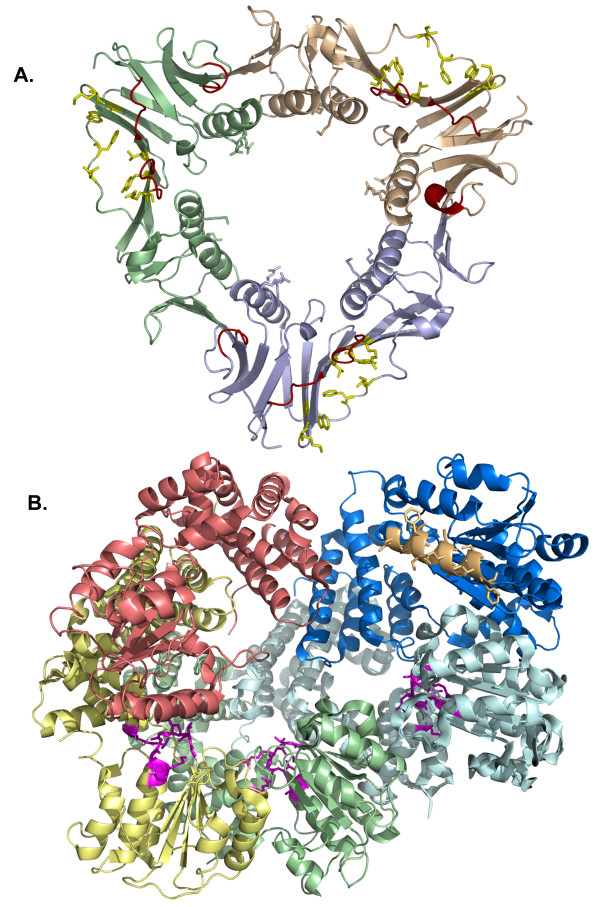
**Structures of T4 gp45 clamp and the E. coli clamp loader, a protein comparable to T4 gp44/62 complex**. A.) The three subunits of the gp45 clamp form a ring with the large opening lined with basic residues which interact with duplex DNA. The binding pocket for interacting with PIP box peptides is shown in yellow. B.) The E. coli γ complex is shown with the γ_3 _subunits (yellow, green, and cyan), the δ' stator subunit (red), and the δ wrench subunit (blue). Also indicated are the regions of the *E. coli *γ complex which interact with the *E. coli *β clamp (orange) and the P-loop motifs for ATP binding (magenta).

The gene 45 protein (gi:5354263, NP_049666), a 228 residue protein, is the polymerase-associated processivity clamp, and is a functional analog to the β subunit of *E. coli *Pol III holoenzyme and the eukaryotic proliferating cell nuclear antigen (PCNA) [8]. All proteins in this family, both dimeric (*E. co*li β) and trimeric (gp45, PCNA), form a closed ring represented here by the structure of the T4 gp45 (PDB 1czd, Figure 4A) [29]. The diameter of the central opening of all known clamp rings is slightly larger than duplex B-form DNA. When these clamps encircle DNA, basic residues lining the rings (T4 gp45 residues Lys 5 and 12, Arg 124, 128, and 131) interact with backbone phosphates. The clamps have an α/β structure with α-helices creating the inner wall of the ring. The anti-parallel β-sandwich fold forms the outer scaffolding. While most organisms utilize a polymerase clamp, some exceptions are known. For example, bacteriophage T7 gene 5 polymerase sequesters *E. coli *thioredoxin for use as a processivity factor [42].

The gp45 related PCNA clamp proteins participate in many protein/DNA interactions including DNA replication, repair, and repair signaling proteins. A multitude of different proteins have been identified that contain a PCNA interaction protein box (PIP box) motif Qxxhxxaa where x is any residue, h is L, I or M, and a is aromatic [43]. In T4, PIP box sequences have been identified in the C-terminal domain of gp43 DNA polymerase, mentioned above, and in the N-terminal domain of RNase H, discussed below. The C-terminal PIP box peptide from RB69 gp43 DNA polymerase has been co-crystallized with RB69 gp45 clamp protein (PDB 1b8h, Figures 3A and 3C) and allows modeling of the gp45 clamp and gp43 DNA polymerase complex (Figure 3A) [31]. The gp45 clamp trails behind the 43 DNA polymerase, coupled through the gp43 C-terminal PIP box bound to a pocket on the outer surface of the gp45 clamp protein. Within RB69 gp45 clamp protein, the binding pocket is primarily hydrophobic (residues Tyr 39, Ile 107, Phe 109, Trp 199, and Val 217) with two basic residues (Arg 32 and Lys 204) interacting with the acidic groups in the PIP box motif. The rate of DNA synthesis, in the presence and absence of gp45 clamp protein, is approximately 400 nucleotides per second, indicating that the accessory gp45 clamp protein does not affect the enzymatic activity of the gp43 DNA polymerase [6]. More discussion about the interactions between T4 gp43 polymerase and T4 gp45 clamp can be found in Geiduschek and Kassavetis, this series. While the gp45 clamp is considered to be a processivity factor, this function may be most prevalent when misincorporation occurs. When a mismatch is introduced, the template strand releases, activating the 3' to 5' exonuclease activity of the gp43 DNA polymerase. During the switch, gp45 clamp maintains the interaction between the replicase and DNA.

#### Gene 44/62 Clamp Loader

The mechanism for loading of the ring shaped PCNA clamps onto duplex DNA is a conundrum; imagine a magician's linking rings taken apart and reassembled without an obvious point for opening. The clamp loaders, the magicians opening the PCNA rings, belong to the AAA + ATPase family which include the *E. coli *gamma (γ) complex and eukaryotic replication factor C (RF-C) [44,45]. The clamp loaders bind to the sliding clamps, open the rings through ATP hydrolysis, and then close the sliding clamps around DNA, delivering these ring proteins to initiating replisomes or to sites of DNA repair. The gp44 clamp loader protein (gi:5354262, NP_049665) is a 319 residue, two-domain, homotetrameric protein. The N-domain of gp44 clamp loader protein has a Walker A p-loop motif (residues 45-52, **G**T**R**GV**GKT**) [38]. The gp62 clamp loader protein (gi:5354306, NP_049664) at 187 residues, is half the size of gp44 clamp loader protein and must be co-expressed with gp44 protein to form an active recombinant complex [46].

The T4 gp44/62 clamp loader complex is analogous to the *E. coli *heteropentameric γ complex (γ_3_δ'δ) and yeast RF-C despite an almost complete lack of sequence homology with these clamp loaders [46]. The yeast p36, p37, and p40 subunits of RF-C are equivalent to the *E. coli *γ, yeast p38 subunit is equivalent to δ', and yeast p140 subunit is equivalent to δ[47]. The T4 homotetrameric gp44 clamp loader protein is equivalent to the *E. coli *γ_3_δ' and T4 gp62 clamp loader is equivalent to the *E. coli *δ. The first architectural view of clamp loaders came from the collaborative efforts of John Kuriyan and Mike O'Donnell who have completed crystal structures of several components of the *E. coli *Pol III holoenzyme including the ψ-χ complex (PDB 1em8), the β-δ complex (PDB 1jqj) and the full γ complex γ_3_δ'δ (PDB 1jr3, Figure 4B) [48-50]. More recently, the yeast RF-C complex has been solved (PDB 1sxj) [47]. Mechanisms of all clamp loaders are likely very similar, therefore comparison of T4 gp44/62 clamp loader protein with the *E. coli *model system is most appropriate. The *E. coli *γ_3_δ', referred to as the motor/stator (equivalent to T4 gp44 clamp loader protein), binds and hydrolyzes ATP, while the δ subunit, known as the wrench (equivalent to T4 gp62 clamp loader protein), binds to the β clamp (T4 gp45 clamp protein). The *E. coli *γ complex is comparable in size to the *E. coli *β clamp and the two proteins interact face to face, with one side of the β clamp dimer interface bound to the δ (wrench) subunit, and the other positioned against the δ' (stator). Upon hydrolysis of ATP, the γ (motor) domains rotate, the δ subunit pulls on one side of a β clamp interface as the δ' subunit pushes against the other side of the β clamp, resulting in ring opening. For the T4 system, interaction with DNA and the presence of the gp43 DNA polymerase releases the gp45 clamp from the gp44/62 clamp loader. In the absence of gp43 DNA polymerase, the gp44/62 clamp loader complex becomes a clamp unloader[6]. Current models of the *E. coli *Pol III holoenzyme have leading and lagging strand synthesis coordinated with a single clamp loader coupled to two DNA polymerases through the τ subunit and to single-stranded DNA binding protein through the χ subunit [51]. There are no T4 encoded proteins that are comparable to *E. coli *τ or χ.

#### Gene 32 Single-Stranded DNA Binding Protein

Single-stranded DNA binding proteins have an oligonucleotide-oligosaccharide binding fold (OB fold), an open curved antiparallel β-sheet [52,53]. The aromatic residues within the OB fold stack with bases, thereby reducing the rate of spontaneous deamination of single-stranded DNA [54]. The OB fold is typically lined with basic residues for interaction with the phosphate backbone to increase stability of the interaction. Cooperative binding of ssb proteins assists in unwinding the DNA duplex at replication forks, recombination intermediates, and origins of replication. The T4 gp32 single-stranded DNA binding protein (gi:5354247, NP_049854) is a 301 residue protein consisting of three domain. The N-terminal basic B-domain (residues 1 - 21) is involved in cooperative interactions, likely through two conformations[55]. In the absence of DNA, the unstructured N-terminal domain interferes with the protein multimerization. In the presence of DNA, the lysine residues within the N-terminal peptide presumably interact with the phosphate backbone of DNA. Organization of the gp32 N-terminus by DNA creates the cooperative binding site for assembly of gp32 ssb filaments[56].

**Figure 5:**
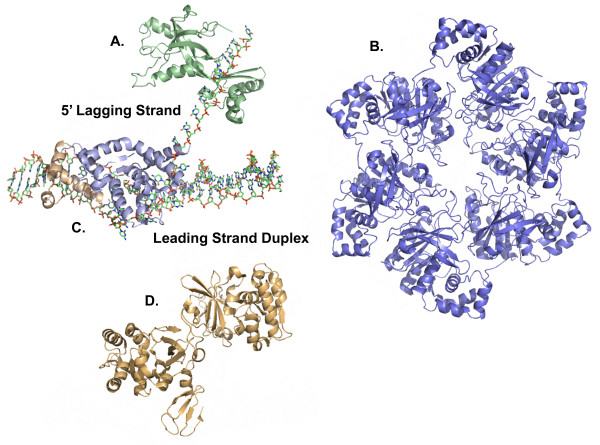
**The T4 primosome is composed of the gp41 hexameric helicase, the gp59 helicase loading protein, the gp61 primase, and the gp32 single stranded DNA binding protein**. A.) the gp32 single-stranded DNA binding protein binds to regions of displaced DNA near the replication fork. B.) the bacteriophage T7 gene 4 helicase domain is representative of the hexameric helicases like the T4 gp41 helicase. ATP binding occurs at the interface between domains. C.) the gp59 helicase loading protein recognizes branched DNA substrates and displaces gp32 protein from the lagging strand region adjacent to the fork. Forks of this type are generated by strand invasion during T4 recombination dependent DNA replication. D.) The two domain ATP dependent bacteriophage T7 DNA ligase represents the minimal construct for ligase activity.

The crystal structure of the core domain of T4 gp32 ssb protein (residues 22 - 239) containing the single OB fold has been solved (Figure 5A) [33]. Two extended and two short antiparallel β-strands form the open cavity of the OB fold for nucleotide interaction. Two helical regions stabilize the β-strands, the smaller of which, located at the N-terminus of the core, has a structural zinc finger motif (residues His 64, and Cys 77, 87, and 90). The C-terminal acidic domain A-domain (residues 240 - 301) is involved in protein assembly, interacting with other T4 proteins, including gp61 primase, gp59 helicase assembly protein, and RNase H [57]. We have successfully crystallized the gp32(-B) construct (residues 21 - 301), but have found the A-domain disordered in the crystals with only the gp32 ssb core visible in the electron density maps (Hinerman, unpublished data). The analogous protein in eukaryotes is the heterotrimeric replication protein A (RPA) [58]. Several structures of Archaeal and Eukaryotic RPAs have been reported including the crystal structure of a core fragment of human RPA70 [59,60]. The RPA70 protein is the largest of the three proteins in the RPA complex and has two OB fold motifs with 9 bases of single-stranded DNA bound (PDB 1jmc). The *E. coli *ssb contains four OB fold motifs and functions as a homotetramer. A structure of the full length version of *E. coli *ssb (PDB 1sru) presents evidence that the C terminus (equivalent to the T4 32 A domain) is also disordered [61].

### Primosomal Proteins

#### Gene 41 Helicase

The replicative helicase family of enzymes, which includes bacteriophages T4 gp41 helicase and T7 gene 4 helicase, *E. coli *DnaB, and the eukaryotic MCM proteins, are responsible for the unwinding of duplex DNA in front of the leading strand replisome [62]. The T4 gp41 protein (gi:9632635, NP_049654) is the 475 residue helicase subunit of the primase(gp61)-helicase(gp41) complex and a member of the p-loop NTPase family of proteins [63]. Similar to other replicative helicases, the gp41 helicase assembles by surrounding the lagging strand and excluding the leading strand of DNA. ATP hydrolysis translocates the enzyme 5' to 3' along the lagging DNA strand, thereby unwinding the DNA duplex approximately one base pair per hydrolyzed ATP molecule. Efforts to crystallize full length or truncated gp41 helicase individually, in complex with nucleotide analogs, or in complex with other T4 replication proteins have not been successful in part due to the limited solubility of this protein. In addition, the protein is a heterogeneous mixture of dimers, trimers and hexamers, according to dynamic light scattering measurements. The solubility of T4 41 helicase can be improved to greater than 40 mg/ml of homogenous hexamers by eliminating salt and using buffer alone (10 mM TAPS pH 8.5) [64]. However, the low ionic strength crystal screen does not produce crystals [65]. To understand the T4 gp41 helicase, we must therefore look to related model systems.

Like T4 41 helicase, efforts to crystallize *E. coli *DnaB have met with minimal success. Thus far only a fragment of the non-hexameric N-terminal domain (PDB 1b79) has been crystallized successfully for structural determinations [66]. More recently, thermal stable eubacteria (*Bacillus *and *Geobacillus stearothermophilis*) have been utilized by the Steitz lab to yield more complete structures of the helicase-primase complex (PDB 2r6c and 2r6a, respectively) [67]. A large central opening in the hexamer appears to be the appropriate size for enveloping single-stranded DNA, as it is too small for duplex DNA. Collaborative efforts between the Wigley and Ellenberger groups revealed the hexameric structure of T7 gene 4 helicase domain alone (residues 261 - 549, PDB 1eOk) and in complex with a non-hydrolyzable ATP analog (PDB 1e0h) [68]. Interestingly, the central opening in the T7 gene 4 helicase hexamer is smaller than other comparable helicase, suggesting that a fairly large rearrangement is necessary to accomplish DNA binding. A more complete structure from the Ellenberger lab of T7 gene 4 helicase that includes a large segment of the N-terminal primase domain (residues 64 - 566) reveals a heptameric complex with a larger central opening (Figure 5B) [69]. Both the eubacterial and bacteriophage helicase have similar α/β folds. The C-terminal Rec A like domain follows 6-fold symmetry and has nucleotide binding sites at each interface. In the eubacterial structures, the helical N-domains alternate orientation and follow a three-fold symmetry with domain swapping. The T4 gp41 helicase is a hexameric two-domain protein with Walker A p-loop motif (residues 197 - 204, **G**VNV**GKS**) located at the beginning of the conserved NTPase domain (residues 170 - 380), likely near the protein:protein interfaces, similar to the T7 helicase structure.

#### Gene 59 Helicase Assembly Protein

The progression of the DNA replisome is restricted in the absence of either gp32 ssb protein or the gp41 helicase [6]. In the presence of gp32 ssb protein, loading of the gp41 helicase is inhibited. In the absence of gp32 ssb protein, the addition of gp41 helicase improves the rate of DNA synthesis but displays a significant lag prior to reaching maximal DNA synthesis [13]. The gp59 helicase loading protein (gi:5354296, NP_049856) is a 217 residue protein that alleviates the lag phase of gp41 helicase [13,14]. In the presence gp32 ssb protein, the loading of gp41 helicase requires gp59 helicase loading protein. This activity is similar to the *E. coli *DnaC loading of DnaB helicase [70,71]. Initially, 59 helicase loading protein was thought to be a single-stranded DNA binding protein that competes with 32 ssb protein on the lagging strand [13,72]. In that model, the presence of gp59 protein within the gp32 filament presumably created a docking site for gp41 helicase. However, the gp59 helicase loading protein is currently known to have more specific binding affinity for branched and Holliday junctions [16,17]. This activity is comparable to the *E. coli *replication rescue protein, PriA, which was first described as the PAS recognition protein (n' protein) in φX174 phage replication [73]. Using short pseudo-Y junction DNA substrates, gp59 helicase loading protein has been shown to recruit gp32 ssb protein to the 5' (lagging strand) arm, a scenario relevant to replication fork assembly [74].

The high-resolution crystal structure of 59 helicase loading protein reveals a two-domain, α-helical structure that has no obvious cleft for DNA binding [17]. The *E. coli *helicase loader, DnaC, is also a two domain protein. However, the C-terminal domain of DnaC is an AAA + ATPase related to DnaA, as revealed by the structure of a truncated DnaC from *Aquifex aeolicus *(pdb 3ec2) [75]. The DnaC N-domain interacts with the hexameric DnaB in a one-to-one ratio forming a second hexameric ring. Sequence alignments of gp59 helicase loading protein reveal an "ORFaned" (orphaned open reading frame) protein; a protein that is unique to the T-even and other related bacteriophages [4,17]. Interestingly, searches for structural alignments of the gp59 protein, using both Dali [76] and combinatorial extension [77], have revealed partial homology with the eukaryotic high mobility group 1A (HMG1A) protein, a nuclear protein involved in chromatin remodeling [78]. Using the HMG1A:DNA structure as a guide, we have successfully modeled gp59 helicase assembly protein bound to a branched DNA substrate which suggests a possible mode of cooperative interaction with 32 ssb protein (Figure 5C) [17]. Attempts to co-crystallize gp59 protein with DNA, or with gp41 helicase, or with gp32 ssb constructs have all been unsuccessful. The 59 helicase assembly protein combined with 32(-B) ssb protein yields a homogenous solution of heterodimers, amenable for small angle X-ray scattering analysis (Hinerman, unpublished data).

#### Gene 61 Primase

The gp61 DNA dependent RNA polymerase (gi:5354295, NP_049648) is a 348 residue enzyme that is responsible for the synthesis of short RNA primers used to initiate lagging strand DNA synthesis. In the absence of gp41 helicase and gp32 ssb proteins, the gp61 primase synthesizes ppp(Pu)pC dimers that are not recognized by DNA polymerase [79,80]. A monomer of gp61 primase and a hexamer of gp41 helicase are essential components of the initiating primosome [63,81]. Each subunit of the hexameric gp41 helicase has the ability to bind a gp61 primase. Higher occupancies of association have been reported but physiological relevance is unclear [82,83]. When associated with gp41 helicase, the gp61 primase synthesizes pentaprimers that begin with 5'-pppApC onto template 3'-TG; a very short primer that does not remain annealed in the absence of protein [79]. An interaction between gp32 ssb protein and gp61 primase likely coordinates the handoff of the RNA primer to the gp43 DNA polymerase, establishing a synergy between leading strand progression and lagging strand synthesis [84]. The gp32 ssb protein will bind to single-stranded DNA unwound by gp41 helicase. This activity inhibits the majority of 3'-TG template sites for gp61 primase and therefore increases the size of Okazaki fragments [6]. Activity of gp61 primase is obligate to the activity of the gp41 helicase. The polymerase accessory proteins, gp45 clamp and gp44/62 clamp loader, are essential for primer synthesis when DNA is covered by gp32 ssb protein [85]. Truncation of 20 amino acids from the C-terminus of gp41 helicase protein retains interaction with gp61 primase but eliminates gp45 clamp and gp44/62 clamp loader stimulation of primase activity [86].

The gp61 primase contains an N-terminal zinc finger DNA binding domain (residues cys 37, 40, 65, and 68) and a central toprim catalytic core domain (residues 179 - 208) [87,88]. Crystallization trials of full length gp61 primase and complexes with gp41 helicase have been unsuccessful. Publication of a preliminary crystallization report of gp61 primase C-terminal domain (residues 192 - 342) was limited in resolution, and a crystal structure has not yet been published [89]. A structure of the toprim core fragment of *E. coli *DnaG primase (residues 110 to 433 of 582) has been solved, concurrently by the Berger and Kuriyan labs (PDB 1dd9, [90]) (PDB 1eqn, [91]). To accomplish this, the N-terminal Zn finger and the C-terminal DnaB interacting domain were removed. More recently, this same DnaG fragment has been resolved in complex with single-stranded DNA revealing a binding track adjacent to the toprim domain (PDB 3b39, [92]). Other known primase structures include the Stearothermophilis enzymes solved in complex with helicase (discussed above) and the primase domain of T7 gene 4 primase (PDB 1nui) (Figure 5D) [69]. The primase domain of T7 gene 4 is comprised of the N-terminal Zn finger (residues 1 - 62) and toprim domain (residues 63 - 255). This structure is actually a primase-helicase fusion protein.

### Okazaki Repair Proteins

#### RNase H, 5' to 3' exonuclease

**Figure 6:**
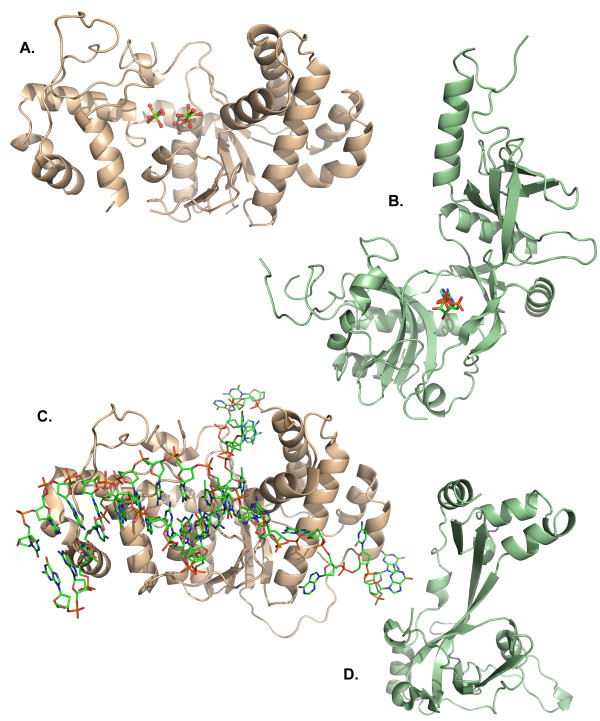
**Lagging strand DNA synthesis requires repair of the Okazaki fragments**. A.) The T4 RNase H, shown with two hydrated magnesium ions (green) in the active site, is a member of the rad2/FEN-1 family of 5' - 3' exonucleases. The enzyme is responsible for the removal of lagging strand RNA primers and several bases of DNA adjacent to the RNA primer which are synthesized with low fidelity by the gp43 DNA polymerase. B.) The T4 DNA ligase, shown with ATP bound in the active site, repairs nicks present after primer removal and gap synthesis by the DNA polymerase. C.) The T4 RNase H structure has been solved with a pseudo-Y junction DNA substrate. D.) The gp32 single stranded binding protein increases the processivity of the RNase H. The two proteins interact between the C-terminal domain of RNase H and the core domain of gp32 on the 3' arm of the replication fork.

RNase H activity of the bacteriophage T4 *rnh *gene product (gi:5354347, NP_049859) was first reported by Hollingsworth and Nossal [24]. The structure of the 305 residue enzyme with two metals bound in the active site was completed in collaboration with the Nossal laboratory (PDB 1tfr) (Figure 6A) [28]. Mutations of highly conserved residues which abrogate activity are associated with the two hydrated magnesium ions [93]. The site I metal is coordinated by four highly conserved aspartate residues (D19, D71, D132, and D155) and mutation of any one to asparagines eliminates nuclease activity. The site II metal is fully hydrated and hydrogen bonded to three aspartates (D132, D157, and D200) and to the imino nitrogen of an arginine, R79. T4 RNase H has 5' to 3' exonuclease activity on RNA/DNA, DNA/DNA 3'overhang, and nicked substrate, with 5' to 3' endonuclease activity on 5' fork and flap DNA substrates. The crystal structure of T4 RNase H in complex with a pseudo Y junction DNA substrate has been solved (PDB 2ihn, Figure 6B) [94]. To obtain this structure, it was necessary to use an active site mutant (D132N); Asp132 is the only residue in RNase H that is inner sphere coordinated to the active site metals [28].

The processivity of RNase H exonuclease activity is enhanced by the gp32 ssb protein. Protein interactions can be abrogated by mutations in the C-terminal domain of RNase H [22] and within the core domain of gp32 ssb protein (Mueser, unpublished data). Full length gp32 ssb protein and RNase H do not interact in the absence of DNA substrate. Removal of the N-terminal peptide of gp32 ssb protein (gp32(-B)), responsible for gp32 ssb cooperativity, yields a protein that has high affinity for RNase H. It is likely that the reorganization of gp32 B-domain when bound to DNA reveals a binding site for RNase H and therefore helps to coordinate 5'-3' primer removal after extension by the DNA polymerase. This is compatible with the model proposed for the cooperative self assembly of gp32 protein. The structure of RNase H in complex with gp32(-B) has been solved using X-ray crystallography and small angle X-ray scattering (Mueser, unpublished data) (Figure 6C). The gp45 clamp protein enhances the processivity of RNase H on nicked and flap DNA substrates [23]. Removal of the N-terminal peptide of RNase H eliminates the interaction between RNase H and gp45 clamp protein and decreases processivity of RNase H. The structure of the N-terminal peptide of RNase H in complex with gp45 clamp protein reveals that binding occurs within the gp45 clamp PIP-box motif of RNase H (Devos, unpublished data).

Sequence alignment of T4 RNase H reveals membership to a highly conserved family of nucleases that includes yeast rad27, rad2, human FEN-1, and xeroderma pigmentosa group G (XPG) proteins. The domain structure of both FEN-1 and XPG proteins is designated N, I, and C [95]. The yeast rad2 and human XPG proteins are much larger than the yeast rad27 and human FEN-1 proteins. This is due to a large insertion in the middle of rad2 and XPG proteins between the N and I domains. The N and I domains are not separable in the T4 RNase H protein as the N-domain forms part of the α/β structure responsible for fork binding and half of the active site. The I domain is connected to the N-domain by a bridge region above the active site which is unstructured in the presence of active site metals and DNA substrate. It is this region that corresponds with the position of the large insertions of rad2 and XPG. Curiously, this bridge region of T4 RNase H becomes a highly ordered *a*-helical structure in the absence of metals. Arg and Lys residues are interdigitated between the active site Asp groups within the highly ordered structure (Mueser, unpublished data). The I domain encompasses the remainder of the larger α/β subdomain and the α-helical H3TH motif responsible for duplex binding. The C-domain is truncated at the helical cap that interacts with gp32 ssb and the PIP motif is located in the N-terminus of T4 RNase H. In the FEN-1 family of proteins, the C-domain, located opposite the H3TH domain, contains a helical cap and an unstructured C-terminal PIP-box motif for interaction with a PCNA clamp.

#### Gene 30 DNA Ligase

The T4 gp30 protein (gi: 5354233, NP_049813) is best known as T4 DNA ligase, a 487 residue ATP-dependent ligase. DNA ligases repair nicks in double-stranded DNA containing 3' OH and 5' phosphate ends. Ligases are activated by the covalent modification of a conserved lysine with AMP donated by NADH or ATP. The conserved lysine and the nucleotide binding site reside in the adenylation domain (NTPase domain) of ligases. Sequence alignment of the DNA ligase family Motif 1 (**K**XDGXR) within the adenylation domain identifies Lys 159 in T4 DNA ligase (159 **K**ADGAR 164) as the moiety for covalent modification [96]. The bacterial ligases are NADH-dependent, while all eukaryotic enzymes are ATP-dependent [97]. Curiously, T4 phage, whose existence is confined within a prokaryote, encodes an ATP-dependent ligase. During repair, the AMP group from the activated ligase is transferred to the 5' phosphate of the DNA nick. This activates the position for condensation with the 3' OH, releasing AMP in the reaction. The T4 ligase has been cloned, expressed, and purified but attempts to crystallize T4 ligase, with and without cofactor, have not been successful. The structure of the bacteriophage T7 ATP-dependent ligase has been solved (PDB 1a0i, Figure 6C) [98,99], which has a similar fold to T4 DNA ligase [100]. The minimal two-domain structure of the 359 residue T7 ligase has a large central cleft, with the larger N-terminal adenylation domain containing the cofactor binding site and a C-terminal OB domain. In contrast, the larger 671 residue E. coli DNA ligase has five domains; the N-terminal adenylation and OB fold domains, similar to T7 and T4 ligase, including a Zn finger, HtH and BRCT domains present in the C-terminal half of the protein [97]. Sequence alignment of DNA ligases indicate that the highly conserved ligase signature motifs reside in the central DNA binding cleft, the active site lysine, and the nucleotide binding site [98]. Recently, the structure of NAD-dependent *E. coli *DNA ligase has been solved in complex with nicked DNA containing an adenylated 5' PO_4 _(pdb 2owo) [101]. This flexible, multidomain ligase encompasses the duplex DNA with the adenylation domain binding to the nick; a binding mode also found in the human DNA ligase 1 bound to nicked DNA (pdb 1x9n) [102]. T4 DNA ligase is used routinely in molecular cloning for repairing both sticky and blunt ends. The smaller two-domain structure of T4 DNA ligase has lower affinity for DNA than the multidomain ligases. The lack of additional domains to encompass the duplex DNA likely explains the sensitivity of T4 ligase activity to salt concentration.

## Conclusion and Future Directions of Structural Analysis

The bacteriophage T4 model system has been an invaluable resource for investigating fundamental aspects of DNA replication. The phage DNA replication system has been reconstituted for both structural and enzymatic studies. For example, the *in vitro *rates and fidelity of DNA synthesis are equivalent to those measured *in vivo*. These small, compact proteins define the minimal requirements for enzymatic activity and are the most amenable to structural studies. The T4 DNA replication protein structures reveal the basic molecular requirements for DNA synthesis. These structures, combined with those from other systems, allow us to create a visual image of the complex process of DNA replication.

Macromolecular crystallography is a biophysical technique that is now available to any biochemistry enabled laboratory. Dedicated crystallographers are no longer essential; a consequence of advances in technology. Instead, biologists and biochemists utilize the technique to compliment their primary research. In the past, the bottleneck to determining X-ray structures was data collection and analysis. Over the past two decades, multiple wavelength anomalous dispersion phasing (MAD phasing) has been accompanied by the adaptation of charge-coupled device (CCD) cameras for rapid data collection, and the construction of dedicated, tunable X-ray sources at the National Laboratory facilities such as the National Synchrotron Light Source (NSLS) at Brookhaven National Labs (BNL), the Advanced Light Source (ALS) at Lawrence Berkeley National Labs (LBNL), and the Advanced Photon Source (APS) at Argonne National Labs (ANL). These advances have transformed crystallography to a fairly routine experimental procedure. Today, many of these national facilities provide mail-in service with robotic capability for remote data collection, eliminating the need for expensive in-house equipment. The current bottle neck for protein crystallography has shifted into the realm of molecular cloning and protein purification of macromolecules amenable to crystallization. Even this aspect of crystallography has been commandeered by high throughput methods as structural biology centers attempt to fill "fold space".

A small investment in crystallization tools, by an individual biochemistry research lab, can take advantage of the techniques of macromolecular crystallography. Dedicated suppliers (e.g. Hampton Research) sell crystal screens and other tools for the preparation, handling, and cryogenic preservation of crystals, along with web-based advice. The computational aspects of crystallography are simplified and can operate on laptop computers using open access programs. Data collection and reduction software are typically provided by the beam lines. Suites of programs such as CCP4 [103] and PHENIX [104,105] provide data processing, phasing, and model refinement. Visualization software has been dominated in recent years by the Python [106] based programs COOT [107] for model building and PYMOL, developed by the late Warren DeLano, for the presentation of models for publication. In all, a modest investment in time and resources can convert any biochemistry lab into a structural biology lab.

What should independent structural biology research labs focus on, in the face of competition from high throughput centers? A promising frontier is the visualization of complexes, exemplified by the many protein:DNA complexes with known structures. A multitude of transient interactions occur during DNA replication and repair, a few of these have been visualized in the phage-encoded DNA replication system. The RB69 gp43 polymerase has been crystallized in complex with DNA, and with gp32 ssb as a fusion protein [36,108]. The gp45 clamp bound with PIP box motif peptides have been used to model the gp43:gp45 interaction [31]. The bacteriophage T4 RNase has been solved in complex with a fork DNA substrate and in complex with gp32 for modeling of the RNaseH:gp32:DNA ternary complex. These few successes required investigation of multiple constructs to obtain a stable, homogeneous complex, therefore indicating that the probability for successful crystallization of protein:DNA constructs can be significantly lower than for solitary protein domains.

### Small angle X-ray and Neutron scattering

Thankfully, the inability to crystallize complexes does not preclude structure determination. Multiple angle and dynamic light scattering techniques (MALS and DLS, respectively) use wavelengths of light longer than the particle size. This allows the determination of the size and shape of macromolecular complex. Higher energy light with wavelengths significantly shorter than the particle size provides sufficient information to generate a molecular envelope comparable to those manifested from cryoelectron microscopy image reconstruction. Small angle scattering techniques including X-ray (SAXS) and neutron (SANS) are useful for characterizing proteins and protein complexes in solution. These low-resolution techniques provide information about protein conformation (folded, partially folded and unfolded), aggregation, flexibility, and assembly of higher-ordered protein oligomers and/or complexes [109]. The scattering intensity of biological macromolecules in solution is equivalent to momentum transfer q = [4π sin θ/λ], where 2θ is the scattering angle and λ is the wavelength of the incident X-ray beam. Larger proteins will have a higher scattering intensity (at small angles) compared to smaller proteins or buffer alone. Small angle neutron scattering is useful for contrast variation studies of protein-DNA and protein-RNA complexes (using deuterated components) [110]. The contrast variation method uses the neutron scattering differences between hydrogen isotopes. For specific ratios of D_2_O to H_2_O in the solvent, the scattering contribution from DNA, RNA, or perdeuterated protein becomes negligible. This allows for the determination of spatial arrangement of components within the macromolecular complex [111]. There are dedicated SAXS beamlines available at NSLS and LBNL. Neutron studies, almost non-existent in the US in the 1990's, have made a comeback with the recent commissioning of the Spallation Neutron Source (SNS) and the High Flux Isotope Reactor (HFIR) at Oak Ridge National Laboratory (ORNL) to compliment the existing facility at the National Institute of Standards and Technology (NIST). The bombardment by neutrons is harmless to biological molecules, unlike high energy X-rays that induce significant damage to molecules in solution.

To conduct a scattering experiment, the protein samples should be monodisperse and measurements at different concentrations used to detect concentration-dependent aggregation. The scattering intensity from buffer components is subtracted from the scattering intensity of the protein sample, producing a 1-D scattering curve that is used for data analysis. These corrected scattering curves are evaluated using programs such as GNOM and PRIMUS, components of the ATSAS program suite [112]. Each program allows the determination of the radius of gyration (R_G_), maximum particle distance, and molecular weight of the species in solution as well as the protein conformation. The 1-D scattering profiles are utilized to generate 3-D models. There are several methods of generating molecular envelopes including *ab initio *reconstruction (GASBOR, DAMMIN, GA_STRUCT), models based on known atomic structure (SASREF, MASSHA, CRYSOL), and a combination of *ab initio*/atomic structure models (CREDO, CHADD, GLOOPY). The *ab initio *programs use simulated annealing and dummy atoms or dummy atom chains to generate molecular envelopes, while structural-based modeling programs, like SASREF, use rigid-body modeling to orient the known X-ray structures into the experimental scattering intensities (verified by comparing experimental scattering curves to theoretical scattering curves). We have used these programs to generate molecular envelopes for the RNaseH:gp32(-B) complex and for the gp59:gp32(-B) complexes. The high resolution crystal structures of the components can be placed into the envelopes to model the complex.

## Abbreviations

ALS: Advanced Light Source; ANL: Argonne National Labs; APS: Advanced Photon Source; BNL: Brookhaven National Labs; CCD: Charge coupled device; DLS: Dynamic light scattering; HFIR: High Flux Isotope Reactor; LBNL: Lawrence Berkeley National Labs; MAD: Multiple wavelength anomalous dispersion; MALS: Multiple angle light scattering; NIST: National Institute for Standards and Technology; NSLS: National Synchrotron Light Source; OB fold: Oligonucleotide-oligosaccharide binding fold; ORNL: Oak Ridge National Laboratory; PCNA: Proliferating cell nuclear antigen; PIP box: PCNA interaction protein box; RF-C: Replication factor - C; SAXS: Small angle X-ray scattering; SANS: Small angle neutron scattering; SNS: Spallation Neutron Source; ssb: single-stranded DNA binding; Toprim: topoisomerase-primase.

## Competing interests

The authors declare that they have no competing interests.

## Authors' contributions

TM was the primary author of this manuscript and created the final constructions of tables and figures. JH contributed the review of scattering methods and assisted in drafting the manuscript. JD created Figures 1 and 2 and assisted in outlining the manuscript. RB created the movies for the supplemental information. KW assisted in drafting the manuscript, provided the expertise in eukaryotic DNA replication and repair, and contributed the majority of editorial assistance. All authors have read and approved the final manuscript.
